# Fibroblasts at the curtain call: from ensemble to principal dancers in immunometabolism and inflammaging

**DOI:** 10.1590/1678-7757-2023-0050

**Published:** 2023-06-23

**Authors:** Rogelio SERRANO-LOPEZ, Ana Carolina MORANDINI

**Affiliations:** 1 Augusta University Dental College of Georgia Department of Oral Biology and Diagnostic Sciences Augusta GA USA Augusta University, Dental College of Georgia, Department of Oral Biology and Diagnostic Sciences, Augusta, GA, USA.; 2 Augusta University Honors Program College of Science and Mathematics Augusta GA USA Augusta University, Honors Program, College of Science and Mathematics, Augusta, GA, USA.; 3 Augusta University Dental College of Georgia Department of Periodontics Augusta GA USA Augusta University, Dental College of Georgia, Department of Periodontics, Augusta, GA, USA.

**Keywords:** Fibroblasts, Stromal cells, Inflammation, Aging, Metabolism

## Abstract

Inflammation is a necessary step in response to injuries, being vital in restoring homeostasis and facilitating tissue healing. Among the cells that play a crucial role in inflammatory responses, stromal cells, including fibroblasts, have an undeniable significance in fine-tuning the magnitude of mediators that directly affect hyper-inflammatory responses and tissue destruction. Fibroblasts, the dominant cells in the gingival connective tissue, are a very heterogeneous population of cells, and more recently they have been receiving well deserved attention as central players and often the ‘principal dancers’ of many pathological processes ranging from inflammation and fibrosis to altered immunity and cancer. The goal of the current investigation is to dive into the exact role of the stromal fibroblast and the responsible mechanistic factors involved in both regulation and dysregulation of the inflammatory responses. This article reviews the most recent literature on how fibroblasts, in their different activation states or subtypes, play a crucial role in contributing to inflammatory outcomes. We will focus on recent findings on inflammatory diseases. We will also provide connections regarding the stromal-immune relationship, which supports the idea of fibroblast coming out from the ‘ensemble’ of cell types to the protagonist role in immunometabolism and inflammaging. Additionally, we discuss the current advances in variation of fibroblast nomenclature and division into clusters with their own suggested function and particularities in gene expression. Here, we provide a perspective for the periodontal implications, discussing the fibroblast role in the infection-driven and inflammatory mediated diseases such as periodontitis.

## Periodontitis: an age-related, infection-driven, and immune-mediated inflammatory disorder

Ageing and age-related diseases share basic mechanistic pathways that ultimately converge to inflammation.^1^ Inflammation has been described as one of the evolutionarily conserved pillars of ageing that are shared by age-related diseases, including metabolic diseases. Inflammaging (or inflammageing) is the term that characterizes the long-term result of chronic physiological stimulation of the innate immune system, which can become degenerative during ageing due to elevated levels of inflammatory markers^2^—a period of life largely unpredicted by evolution.^1^ Inflammation is usually referred as a response to infection or injury, and chronic inflammation is commonly associated with age-related diseases^3^ such as metabolic dyslipidemia, obesity,^4^ cardiovascular,^5^ kidney,^6^ and brain^7^ disorders.

The inflammatory behavior behind some conditions is a normal response, carrying mostly a protective and beneficial role to the body. However, the inflammatory response can become detrimental and exaggerated, especially when it results in tissue destruction, which is directly proportional to the extended duration. Such descriptive scenario is common in patients with many forms of periodontitis, characterized by alveolar bone loss because of untreated gingivitis. A significant body of literature have shown inflammatory responses, such as those found in periodontitis, can be sustained and driven by keystone pathogens, which surprisingly are “inflammo-philic” (= as if attracted to inflammation) as previously described.^8^ *Porphyromonas gingivalis* is a relevant example of keystone pathogen,^9^ which has evolved many strategies to subvert the immune system and successfully thrive in the host while maintaining the disease state.^10 - 13^ Therefore, periodontitis can be considered an infection-driven and immune mediated inflammatory disorder (IMID).

Infection-driven and IMID have shown a rapid increase over the last few decades.^14^ Another example of IMID is Crohn’s disease (CD), a chronic inflammatory process of the digestive tract with the involvement of the tumor necrosis factor-alpha (TNF-α) cytokine. TNF-α is a 157-amino acid pro-inflammatory cytokine, predominantly produced by monocytes, macrophages, and T-cells. It is involved in transcription of genes coding other inflammatory mediators and induction of matrix metalloproteinases.^15^ Intestinal mucosa samples from patients with CD showed high levels of TNF-α,^16^ indicating a dysregulation of this cytokine similar to dysregulations found in other IMID, such as rheumatoid arthritis, cutaneous inflammatory conditions, and connective tissue disorders.^17^

The physiological steady state is usually maintained by homeostatic mechanisms, which are tightly managed by the nervous and endocrine systems. This leaves the host immune system as the inflammatory control mechanism to eliminate noxious stimulus such as infection or tissue damage.^3^ Interestingly, in this case there is no “Which came first? – the chicken or the egg-story”. The selective flourishing of inflammophilic bacteria can perpetuate inflammatory tissue destruction by fueling a vicious cycle for disease progression, in which periodontal dysbiosis and inflammation reinforce and fuel each other.^8^ One of the main players in this dysbiotic game is the keystone pathogen *P. gingivalis* , which can frequently be detected at low levels in the “normal” periodontal microbiota of healthy individuals and has a community-wide impact, which involves host modulation and disease pathogenesis.^9^ *P. gingivalis* and its virulence factors have been extensively studied in consortium with other species characterizing a polymicrobial synergy,^18^ which is directly associated to alveolar bone loss.^19^ In this aspect, many cell types are important to orchestrate tissue response and bridge the link to the overall systemic health, including antigen-presenting cells,^20^ T cells,^21 , 22^ and architectural stromal cells, which can assume an immune phenotype and function on demand.^23^

## The role of fibroblasts in inflammation

Fibroblasts have long been part of the ‘ensemble’ of cells, being the dominant group of cells mainly performing a structural role in the stroma. Their role is intimately linked to the extracellular matrix and, to a large extent, they are responsible for the synthesis of the fibrillar constituents of the stroma. In addition, they regulate the function of adjacent epithelial cells via bidirectional interaction and secretion of growth factors and cytokines.^24^ Recently, they have been receiving deserved attention as “protagonists” of the cell-cell communication, orchestrating the stromal-immune cell relationships and controlling the abundance of inflammatory cytokines and chemokines, which directly affects the inflammatory microenvironment and tissue outcomes.

We and others have shown that fibroblasts play an active role in oral inflammation^25 - 27^ and fibrosis.^28^ Their inflammatory profile is typified by hypersecretion of cytokines, such as Interleukin (IL)-6, and inflammatory chemokines, such as CXCL8 and CXCL12 in a different manner depending on their site of origin.^29^ Cytokines are small proteins produced and released with the final aim of cell-cell communication. These signaling molecules are then responsible for the autocrine, paracrine, and endocrine activities and play an immunomodulation function. After binding to specific receptors on various types of cells, cytokines induce activation, proliferation, or migration of target cells. Additionally, there are several categories of cytokines, including interleukins and chemokines^30^ with considerable redundancy. Numerous studies highlight the association of cytokine activity and fibroblast response in human periodontitis. Although fibroblasts decreased tumor necrosis factor (TNF)-α secretion, they were shown to enhance the ability of macrophages to phagocytose bacteria.^31^ In the same study, gingival fibroblasts, which were called fibroblasts from peri-implantitis inflamed tissues, were at least as active as periodontal ligament fibroblasts in regulating macrophage responses to bacteria.^31^ Macrophage migration inhibitory factor (MIF) is recognized as a cytokine involved in macrophage and T-cell activation, cell growth, apoptosis, tumor angiogenesis, and carbohydrate metabolism. CD74 is the receptor with direct activity and recruitment occurs mainly via CXCR2 and CXCR4 signaling. Once in circulation, MIF and lipopolysaccharide (LPS) were found to regulate the release of pro-inflammatory cytokines such as TNF-α, IL-1β, IL-8/CXCL8, Cyclooxygenase-2 (COX-2), Nitric oxide, and products of arachidonic acid pathway.^32^

Additionally, fibroblast sensitivity toward periodontal pathogens reinforces the infection-driven and inflammatory-mediated character of periodontitis. Uncontrolled secretion of large number of cytokines during sepsis or infection is referred to as “cytokine storm” in chronic inflammatory conditions and, in this context, MIF can be described as a key player among other cytokines.^32^ The effects of MIF on fibroblast migration in wounded monolayers *in vitro* was previously investigated. Transient but not permanent exposure of primary mouse or human fibroblasts with MIF significantly promoted wound closure, a response that encompassed both a proliferative and a pro-migratory component.^33^ *P. gingivalis* was found to contribute to the development of periodontitis via MIF.^34^ A study has compared the expression of MIF and CCL2/MCP-1 (Monocyte chemoattractant protein-1), one of the most studied chemokines, as it takes part in immune surveillance and immune modulation. It was found that the level of MIF was elevated in the serum and in the gingival tissue of individuals with periodontitis compared with healthy subjects, similarly to the levels of CCL2/MCP-1.^34^

Another key chemokine abundantly produced by stimulated fibroblasts is CXCL8 chemokine, which is a hallmark for neutrophilic attraction to the inflammatory site. Excessive expression of CXCL8 arrests the wound in the inflammatory phase, and is associated with nonhealing chronic inflammation in models of diabetic foot ulcer.^35^ A recent report suggests that continued cytokine secretion in the wound mediates changes in fibroblast phenotype and causes arrest in the inflammatory phase without progressing to the resolution phase. Thus, limiting CXCL8 secretion would attenuate inflammation as well as decrease secretory fibroblast phenotype and enhance wound healing.^35^ In our previous work with gingival fibroblasts *in vitro* , we showed that fibroblasts are hyper-activated with IL-1β stimulus, secreting large amounts of CXCL8, which can be controlled through activation of adenosine receptors.^36^ Ongoing studies in our lab are focused on unveiling the mechanisms of suppression of hyper-inflammatory state of gingival fibroblasts and our preliminary data suggest mitochondrial metabolism is central to this plasticity (unpublished results).

Relatively limited research has been conducted on the interactions of stromal-immune cell participating in inflammation behind periodontitis when compared to other diseases such as rheumatoid arthritis (RA)^37^ or cancer.^38^ Cancer-activated fibroblasts interact with myeloid cell-derived immune cells in the tumor microenvironment to enhance tumorigenesis and immune evasion.^38^ Notably, it has been suggested that each stromal cell developed in the tumor-specific microenvironment has a ‘dual’ pro-tumorigenic or anti-tumorigenic role depending on the interaction with immune cells,^38^ increasing the complexity of these interactions.

On the other hand, RA is notorious for alternating state of inflammation of non-active disease or between active and silent periods. A previous study explored the association of synovial fibroblasts (SF) and their metabolic reprogramming and inflammasome activation *in vivo.* ^39^ In addition to fibroblasts direct relationship with macrophages in response to the inflamed joints, SF gradually acquired a more disease-promoting phenotype during arthritis, characterized by the production of inflammatory mediators^39^ such as the subset of the SF itself. This study found Thy1+CD34+ as SF subset, which interacts with vascular endothelial cells. This characterizes SF as a proliferative and invasive cell behavior, triggering tissue destruction and promoting leukocyte trafficking into joints.^39^ Interestingly, the same study demonstrated SF altered cellular metabolic pathways towards glycolysis. In this particular case, hexokinase 2 was suggested as a possible point of interest as intervention for the altered function state of SF.^39^ Therefore, it seems that the inflammatory or anti-inflammatory state or phenotype of the fibroblast as stromal cells can be highly influenced by their metabolic state, which started to be further investigated more recently.

## The role of fibroblasts in Immunometabolism

Traditionally, immunology and metabolism have been considered distinct disciplines. However, a significant body of evidence regarding the intersection between immune response and the interference of metabolic signaling pathways on immune cells and stromal cells, such as fibroblasts, has brought upon a new term to the field, known as immunometabolism.^40^ Recent research suggests the existence of interactions between the metabolic and immune systems controlling pathogenic mechanisms, which may underlie many of the downstream complications, especially in chronic inflammatory diseases. For example, obesity affects the immune system and promotes inflammation, which in return promotes a variety of chronic conditions and diseases.^41^ This supports leukocytes and lymphocytes behavior on several levels, which are controlled by internal metabolic processes. Additionally, immune cells use and respond to nutrients similarly to other cells. This applies to scenarios other than obesity. An example of this is the serine/threonine kinases AKT (AKT serine/threonine kinase), AMPK (AMP-activated protein kinase), mTOR (mammalian target of rapamycin), and LKB1 (liver kinase B1). These are generally thought of as cellular nutrient sensors that help to maintain energy homeostasis by relaying signals that determine how cells respond to high or low levels of intracellular carbohydrate and amino acids.^40^

In tumor microenvironment, metabolic flux—including deprivation of metabolic substrates, accumulation of metabolic waste, and metabolism activity in various types of cell—has crucial effects on the antitumor response.^42^ In a previous *in vivo* study, Chondroitin-6-Sulfate–targeted strategies decreased M2 macrophages and reprogrammed the immunosuppressive tumor microenvironment, leading to enhanced response to anti-programmed cell death protein (PD)-1 in colorectal cancer.^43^ Additionally, glycogen lies at the nexus of diverse processes that promote malignancy, including proliferation, migration, invasion, and chemoresistance of cancer cells. Thus, approaches dedicated on targeting glycogen metabolism in cancer represent a promising, yet under-explored, therapeutic avenue. For example, in macrophages, acute inflammation causes glycogen mobilization as a feedstock for the pentose phosphate pathway and Nicotinamide adenine dinucleotide phosphate (NADPH) production.^44^ Glycogen also drives the initial stages of dendritic cell activation via glycolytic reprogramming.^45 , 46^ These physiological processes are believed to contribute to cancer progression.

Similarly, there has been an increasing demand for studies on immunometabolism and periodontitis. A previous investigation explored the role of metabolic disturbance in immunoregulation of gingivitis targeting T helper 17 (Th17)/regulatory T cells (Treg). As a result, the percentages of CD4, IL17A, and Th17 cells significantly increased in the peripheral blood in the gingivitis group in comparison with the healthy group. Furthermore, the study identified 18 different metabolites, which were differentially expressed in plasma between the gingivitis and the healthy control groups. Notably, the levels of cholesterol, glycerol 1-octadecanoate, d-glucose, uric acid, cyclohexane acetic acid, 3-pyridine, tryptophan, and undecane 2,4-dimethyl were significantly up-regulated.^47^

Stromal cells are the dominant cell type of connective tissues throughout the body and provide architecture for the support of functional cells in various systems. A study combining single cell RNA-sequencing and FACS in adult mice found visceral adipose tissue (VAT) as an endocrine organ that plays a key role in organismal homeostasis by integrating metabolic and immunological aspects.^48^ Cell-cell interactions highlight the pivotal role of distinct subtypes of mesenchymal stromal populations as orchestrators of metabolic homeostasis,^48^ namely adipogenic visceral adipocyte precursor cells and fibro-inflammatory progenitors. In healthy state, fat deposit participates in the storage and release of lipids as per physiological demand while maintaining a local anti-inflammatory environment, as reviewed elsewhere.^49^ Adipocytes play a role to sustain adequate lipid storage and immune regulation via crosstalk with specialized tissue-resident immunocytes, especially Tregs^50^ and group 2 innate lymphoid cells (ILC2s)^51^ to prevent the development of local inflammation.

## The versatility of fibroblasts as protagonists of inflammation and active players of inflammaging

**Figure 1:**
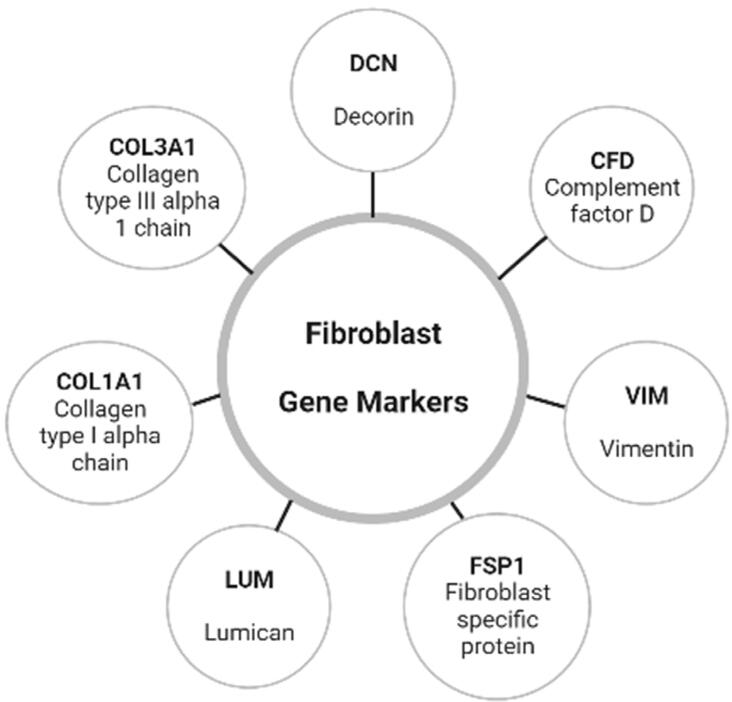
Diagram representing the most common genes used as markers for human gingival fibroblasts. Created with Biorender

Stromal cells have been predominantly focused on inflammatory diseases research. Evidence of different subsets of fibroblasts, which was unprecedented until very recently, have been revealed in different models. The article by Williams, et al.^52^ (2021) defined five subsets of fibroblasts and referred to them as fibroblast clusters; however, only four subsets were interpreted as primarily different between healthy and periodontitis, namely: Fib1.1 (CXCL1, CXCL2, CXCL13); Fib 1.2 (APCDD1, IGFBP2, MRPS6); Fib1.3 (APOD, GSN, CFD); and Fib1.4 (TIMP3, ASPN, COL11A1). This was arguably one of the most in-depth studies of fibroblast atlas of human oral mucosa in individuals with and without periodontitis, although with some limitations such as the small sample size and no insights regarding sex or age differences. In addition, phenotypic markers, such as Collagen type I, Decorin, among other common markers of fibroblasts, were applied to separate fibroblast population from epithelial, endothelial, immune, and other cell types. Although not exclusively expressed on fibroblasts, vimentin (VIM)^53^ and fibroblast specific protein 1 (FSP1, S100A4)^54^ have served as useful markers to characterize fibroblasts by immunostaining approaches. We and others have characterized the fibroblastic phenotype *in vitro* by the expression FSP1.^26 , 54 - 57^ Figure 1 summarizes the most common fibroblast markers, including more recent ones derived from single cell transcriptome studies.

**Figure 2:**
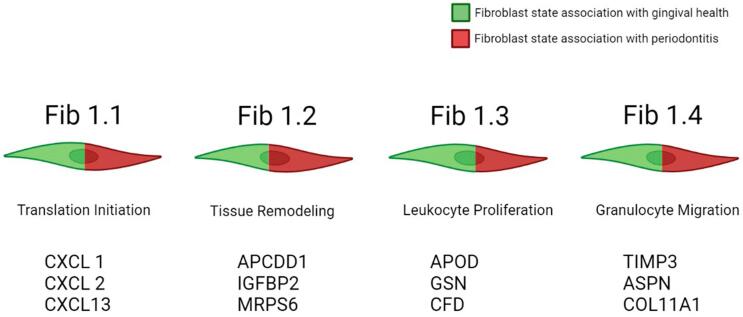
Gene markers and signatures mostly associated with each fibroblast cluster according to Williams et al.52 Each fibroblast state was clustered and compared according to the percentage of positive cells in healthy and periodontitis conditions. Created with Biorender

According to Williams, et al.^52^ (2021) the clusters Fib1.2 and Fib1.3 represented the most differently expressed between health and periodontitis states, although not significantly. Figure 2 shows the main genes representing each fibroblast cluster regarding different gene-expression levels between healthy and periodontitis. The authors also identified five unique subsets of functionality. Fibroblast cluster 1 shows high expression of genes involved in active translation, with abundant expression of ribosomal proteins, whereas fibroblast cluster 2 displays a gene signature corresponding to the prototypic functions of the cell type, including collagen synthesis and tissue remodeling. Fibroblast clusters 3, 4, and 5 express gene signatures consistent with inflammatory responses, such as regulation of leukocyte proliferation, granulocyte migration, and complement activation, respectively. Some of these clusters have direct association with neutrophils and pro-inflammatory cytokines, therefore suggesting that periodontal tissue immunity depends on the strong stromal-neutrophilic interaction within these tissues.

A recent article applying rheumatoid arthritis *in vivo* model found distinct subsets of fibroblasts responsible for mediating either inflammation or tissue damage in rheumatoid arthritis. The experiments involved tampering with the fibroblast activation protein-α (FAPα). More specifically, FAPα+THY1- fibroblasts selectively mediate bone and cartilage damage with insignificant effect on inflammation, whereas transfer of FAPα+ THY1+ fibroblasts resulted in a more severe and persistent inflammatory arthritis with minimal effect on bone and cartilage.^58^

Perhaps the involvement of different subsets present in arthritis should be cautiously interpreted when trying to apply to other diseases. A similar type of study was conducted in a model of the human eye-atopic dermatitis (AD). The *in vitro* study found that fibroblasts demonstrated a novel COL6A5+COL18A1+ subpopulation that was unique to lesional AD and expressed CCL2 and CCL19 chemokines. The method included single-cell RNA sequencing on skin biopsy specimen both lesional and nonlesional sample along with a control. Findings concluded AD lesions were characterized by expanded type 2/type 22 T cells and inflammatory DCs, and by a unique inflammatory fibroblast that may interact with immune cells to regulate lymphoid cell organization and type 2 inflammation.^59^ Type 2 inflammatory response involves T helper cells, which release cytokines such as IL-4, IL-5, IL-9, and IL-13. Type 2 immunity shows many host-protective functions, including maintenance of metabolic homeostasis, suppression of excessive type 1 inflammation, maintenance of barrier defense, and regulation of tissue regeneration, as previously published in a review^60^. Nonetheless, excessive and chronic activation of the type 2 response can lead to allergic disease and reduction in pathogen defense.^60^

Recent studies^61 , 62^ have been consistently showing that the role of stromal cells, such as fibroblasts, is beyond being the most numerous resident cells functioning as a group, or an ‘ensemble’ in a particular extracellular matrix or tissue. The new perspective of fibroblasts as protagonists or, in our analogy, ‘principal dancers’ of inflammatory disorders^62^ is sustained by the presence of the different subset populations from within the cell type with an important role revealed as shared pathological activation states across chronic inflammatory diseases.^61^ This begs the question: what are the roles of these different subsets and how can they change depending on environmental conditions? Further research is necessary to define and to identify these subsets, respecting the individuality of each tissue type. Future studies should provide further clues on the participation of these cells in different diseases of inflammaging, including periodontitis.

Recent data by Caetano, et al.^63^ (2023) combining multi-omics techniques and fluorescence *in situ* hybridization suggest the emergence of a spatially restricted pathogenic fibroblast population, expressing CXCL8 and CXCL10, referred as fibroblast 5 (characterized by RAC2 and LCP1 markers). This spatially restricted population in the gingival lamina propria would be responsible for neutrophil and lymphocyte recruitment at periodontal pocket sites, and, with angiogenic properties, it could be responsible for irregular chemokine gradients in the development of persistent inflammation characteristic of periodontitis. In a previous report, Caetano, et al.^64^ (2021) compared scRNA-seq data from four individuals presenting progressive states from healthy, mild, and severely diseased. In addition to identifying five fibroblast-like populations (Fibroblast 1 through 5), one pericyte, and one myofibroblast population, the authors reported the genes IL1B, EDN1, TNF, and BMP2 as the main epithelial modulators driving an inflammatory response in stromal and perivascular cells. Based on their expression, the authors provided a platform of epithelial-mesenchymal interactions in periodontitis, especially between epithelial IL1B and TNF and stromal target genes. ^64^

Age-related changes affecting neutrophils, macrophages, and T cells seem to promote a pathogenic immune response and contribute to the increased prevalence of periodontitis in older adults.^65^ Fibroblasts from aged donors (50–70 year old) showed a significant decrease in cell proliferation, migration, activation, and collagen remodeling when compared with young-donors’ (15–25 years old) derived primary fibroblasts.^66^ In addition, evidence for functional specialization of human dermal fibroblasts identified partial loss of their cellular identity and expression of skin aging-associated secreted proteins as an important age-related change in human skin. Authors demonstrated substantial reduction in the interactions between dermal fibroblasts and undifferentiated keratinocytes at the dermal-epidermal junction.^67^ Additionally, human dermal fibroblasts from donors of different ages were used as a model to study how single cell migration, biophysical, and morphological properties are altered by donor age.^68^ Authors demonstrated that donor aging resulted in reduction of cell motility and increased amounts of F-actin, tubulin, and dominantly vimentin.^68^

Fibroblast transcriptional states are conserved between mice and humans, including universal fibroblasts and activated phenotypes associated with pathogenicity in human cancer, fibrosis, arthritis, and inflammation.^69^ This includes characterization of fibroblast in both healthy and disease states in different organs. Immunological properties of fibroblasts have been reported by findings that characterize fibroblasts as directors of leukocyte behavior such as promoting the secretion of type-I IFNs to sustain survival of differentiated T cells.^70^ The evidence of site-specific stromal regulation, which was later defined as stromal address code within different tissues, was previously reviewed by Parsonage, et al. ^71^ (2005).

The increasingly appreciated heterogeneity of fibroblast identities and properties across different organs^72^ and within single tissues^63^ has recently started to unravel, driven by advances in single-cell transcriptomic and proteomic methodologies. This heterogeneity commonly reflects their origin and relative location in the tissue microenvironment, as well as the nature of their interactions with neighbor cells. Therefore, there is indication that they have adapted to have tissue-specific functions and enough plasticity within the same tissue to behave according to the microenvironment demand and assume inflammation-associated identity.

## Final considerations

Recent advances in single-cell technology approaches contributed to better understand the complexity of the previous considered ‘ensemble’ and architectural cells. The versatility of fibroblasts depending on the tissue microenvironment is intriguing and deserves more studies to unveil the exact role of different subsets or phenotypes in immune-mediated inflammatory diseases. Whether these are truly different populations or different states of activation of the cells, which may vary depending on the tissue type and biological ageing, remains unclear. Their protagonist role is fascinating and reveals the beauty of the stromal immunology as a rising star in the biological processes of immune-mediated inflammatory diseases.
